# Supplementary material for: A high-throughput assay for the measurement of Ca2+ oscillations and insulin release from uniformly sized mouse β-cell (MIN6) spheroids
Source: eLife. 2026 Oct 5;15:RP110428. doi: 10.7554/eLife.110428 (PMC13638198; doi:10.7554/eLife.110428)
Supplement: Peer review history

# Editor report: eLife Assessment

**DOI:** 10.7554/eLife.110428.3.sa0

**Authors:** Leon D Islas

**Affiliations:** Universidad Nacional Autónoma de México Mexico

**Keywords:** Solid, Valuable

In this **valuable** study, Robben et al. describe a 3D beta-cell spheroid culture platform, that allows high-throughput monitoring of cytoplasmic calcium concentrations and insulin secretion. The authors demonstrate measurements of calcium signals comparable to those recorded in primary pancreatic islets. The authors validate the method by culturing MIN6 cells in a 3D-culture system, and show **solid** evidence of its utility by recording calcium signals in a high-throughput format and characterizing these calcium signals using pharmacological tools. This methodology demonstrates the utility of the 3D beta-cell spheroids for screening pharmacological modulators of pancreatic beta-cell function.

# Referee report: Reviewer #1 (Public review)

**DOI:** 10.7554/eLife.110428.3.sa1

[Editors' note: this version has been assessed by the Reviewing Editor without further input from the original reviewers and the authors have satisfactorily answered the previous reviewer's comments.]

Summary:

They use cultures of insulinoma MIN6 cells that form spheroids in a micro-patterned PEG-hydrogel to measure Ca2+ oscillations in multiple cells simultaneously.

Strengths:

They demonstrate that insulinoma spheroids are formed in multi-well plates and that Ca2+ imaging can be performed on them.

# Referee report: Reviewer #2 (Public review)

**DOI:** 10.7554/eLife.110428.3.sa2

Summary:

The study by Robben et al., show 3D beta-cell spheroid platform, a valuable tool allowing high-throughput monitoring of cytoplasmic Ca concentrations and insulin secretion, with Ca signals comparable to those recorded in primary islets. The authors demonstrate a solid method to culturing MIN6 cells in a 3D culture system, recording Ca signals in a high-throughput format and characterizing these Ca signals using pharmacological tools, including TRPM3 channel and K-ATP channel modulators. This highlights the utility of the 3D beta-cell spheroid for screening new ion channel modulators in beta-cells of the pancreas.

Strengths:

- The study shows that the MIN-6-based 3D beta-cell model is better to study Ca-signaling and insulin secretion compared to 2D culture of single MIN-6 cells.

- The method allows imaging of Ca signaling in many spheroids in parallel followed by collecting medium to measure insulin release and correlate both effects.

- The authors demonstrate that this system is suitable for screening new pharmacological modulators and used as an agonist of the ATP-sensitive potassium channel (diazoxide) and the agonist and antagonist of the TRPM3 channel.

# Referee report: Reviewer #3 (Public review)

**DOI:** 10.7554/eLife.110428.3.sa3

Summary:

The primary objective of this study is to develop high-throughput screening assays utilizing homogeneous 3D cell cultures that more accurately replicate the intricate architecture and cellular communication found in tissues. The authors have chosen pancreatic islet β-cells as a model system to evaluate agents that modulate insulin release, which is particularly relevant given the increasing prevalence of diabetes mellitus-a significant global health concern. Moreover, the incorporation of human-based 3D spheroids, organoids, or organ-on-chip technologies into drug discovery protocols is essential for enhancing clinical translation, as candidate compounds identified using animal models have often demonstrated limited success in clinical settings.

Strengths:

This study was thoughtfully planned and skillfully carried out. The use of micropatterned hydrogels to observe 19 spheroids at once is an ingenious aspect, which has been effectively validated with Ca microfluorography. Overall, I found this investigation to be exceptionally well-executed and free from notable flaws, as the results clearly back up the conclusions. Additionally, the developed method achieved the proposed aims, providing a high-throughput format with 3D cultures. I believe this study deserves publication.

# Author comment: Author response

**DOI:** 10.7554/eLife.110428.3.sa4

**Authors:** Stijn Robben, Patricia Davidson, Rita S Rodrigues Ribeiro, Thomas Voets

**Affiliations:** VIB-KU Leuven Center for Neuroscience Leuven Belgium; 4Dcell Montreuil France; 4Dcell Montreuil France; VIB-KU Leuven Center for Neuroscience Leuven Belgium

The following is the authors’ response to the original reviews.

**Reviewer #1 (Public review):**

Summary:

They use cultures of insulinoma MIN6 cells that form spheroids in a micro-patterned PEG-hydrogel to measure Ca ^2+^ oscillations in multiple cells simultaneously.

Strengths:

They demonstrate that insulinoma spheroids are formed in multi-well plates and that Ca ^2+^ imaging can be performed on them.

Weaknesses:

The type of equipment and multi-wells used for the experiments are very specialized to be used as a common tool. Insulinoma cells are tumoral cell lines that divide, unlike primary beta cells. Pancreatic islets are very different from this preparation, as they are highly heterogeneous, whereas these cells all respond equally. It would be good to see the same technique applied to primary cells.

MIN6 cells do not respond to glucose and other secretagogues in the same way as primary cells, and they cycle, depending on the phase of the cycle to which they are exposed.

The authors should report the number of cells per spheroid and the number of cells that are alive and dead.

I would like to examine the effects of calcium channel blockers on calcium transients, and the use of pregnenolone is already described in the literature, but remains less well known.

MIN6 cells secrete much insulin, because detecting the hormone in ELISAs requires too many primary cells. The authors should discuss the model in greater detail and compare it with primary beta cells. Also, they take 3 mM glucose as the basal concentration, which is low.

We thank the reviewer for their valuable comments, which have helped us to significantly enhance the quality of our manuscript. We have carefully considered these comments and have revised the manuscript accordingly. A point-by-point rebuttal is provided below.

**Reviewer #1 (Recommendations for the authors):**

(1) The manuscript contains numerous typos, including the combination of numbers and units without a space.

All spacing inconsistencies between numerical values and unit symbols (e.g. mM, μM, µL, and Hz) have been corrected throughout the text and images. In addition, the following typographical and grammatical errors have been addressed:

- Abstract: "the frequency of Ca ^2+^ oscillations correlate" → correlates

- Figure 2A caption: "200uL" → 200 µL

- Figure 5A caption: "glimepirde" → glimepiride

- Figure 5 title: "KATP-antagonists induces" → induce

- Figure 7D caption: "concentrations" → concentration

- Figure 7 caption: "Tukey’s post-hoc testm" → Tukey’s post-hoc test

- Results section header: "increases insulin secretions" → insulin secretion

- Discussion: "we obtained an EC50 values 7.4 ± 0.4 mM" → "we obtained EC50 values of 7.4 ± 0.4 mM"

- Discussion: "a EC50 value" → an EC50 value

- Discussion: "concentration dependence profiles that matches" → match

- Discussion: "PS-induced activation TRPM3" → activation of TRPM3

- Conclusion: "found the Islets of Langerhans" → found in the Islets of Langerhans

- Acknowledgements: "grant agreement No. 955643" → agreement No. 955643

(2) I suggest showing the experiments in primary beta cells because of the many differences from insulinoma cells, as the most important result is a better culture technique for calcium imaging.

We thank the reviewer for this valuable suggestion. We did indeed attempt to perform comparable experiments using the Cellartis hiPS Beta Cell Media Kit (Takara, cat. no. Y10108) as a more physiologically relevant cell model. To minimize cellular stress during the transition, we adjusted our protocol by seeding the differentiated cells into the micropatterned plates 24 hours prior to imaging, thereby maintaining the recommended culture conditions for as long as feasible. However, during calcium imaging, the cells failed to respond to either the elevated glucose stimulus or the positive control, suggesting that the cells did not survive the transfer to our plate format with sufficient viability to mount a functional response.

We acknowledge that the use of primary beta cells or hiPS-derived beta cells would strengthen the physiological relevance of the platform. Nevertheless, we would like to emphasize that the primary objective of this study is to demonstrate the feasibility of high-throughput calcium imaging in 3D cell culture using our optimized protocol: a proof-of-concept that is, by design, independent of the specific cell model employed. Adapting the protocol to accommodate more sensitive or terminally differentiated cell types is a meaningful avenue for future work, but falls outside the scope of the current manuscript. We have added a brief note to the Discussion section to explicitly acknowledge this limitation and to identify hiPS-derived beta cell compatibility as a priority for subsequent optimization.

(3) These kinds of cultures in three dimensions are interesting, but it has been shown that it is even better to have the liquid flow, simulating blood flow; this can at least be discussed.

We thank the reviewer for this insightful comment. We agree that the introduction of perfusion-based flow represents a meaningful improvement over static 3D culture systems. We have incorporated this point into the Discussion section, where we now explicitly acknowledge the potential benefits of dynamic culture conditions, including improved viability, insulin secretory function, and morphological integrity of 3D β-cell tissues, and identify the integration of perfusion-induced flow as a potentially meaningful path to explore for future development of the platform.

**Reviewer #2 (Public review):**

Summary:

The study by Robben et al., show 3D beta-cell spheroid platform, a valuable tool allowing high-throughput monitoring of cytoplasmic Ca concentrations and insulin secretion, with Ca signals comparable to those recorded in primary islets. The authors demonstrate a solid method to culturing MIN6 cells in a 3D culture system, recording Ca signals in a high-throughput format and characterizing these Ca signals using pharmacological tools, including TRPM3 channel and K-ATP channel modulators. This highlights the utility of the 3D beta-cell spheroid for screening new ion channel modulators in beta-cells of the pancreas.

Strengths:

- The study shows that the MIN-6-based 3D beta-cell model is better to study Ca-signaling and insulin secretion compared to 2D culture of single MIN-6 cells.

- The method allows imaging of Ca signaling in many spheroids in parallel followed by collecting medium to measure insulin release and correlate both effects.

- The authors demonstrate that this system is suitable for screening new pharmacological modulators and used as an agonist of the ATP-sensitive potassium channel (diazoxide) and the agonist and antagonist of the TRPM3 channel.

Weaknesses:

- The study is based on only one cell line, the MIN6 insulinoma cells, which may not fully mimic the pancreatic beta-cells within the islet.

- The authors show only spheroids cultured overnight. A long-term culture is missing to assess beta-cell viability long term function.

- The authors tested their platform using only two compounds. Testing a larger compound library is necessary to make a clear conclusion about the suitability of the platform for high-throughput screening.

We thank the reviewer for their valuable comments, which have helped us to significantly enhance the quality of our manuscript. We have carefully considered these comments and have revised the manuscript accordingly. A point-by-point rebuttal is provided below.

**Reviewer #2 (Recommendations for the authors):**

Major Points

(1) In this study, only 2 pharmacological compounds (for TRPM3, and K channel) were tested. Testing a larger compound library would be necessary to fully demonstrate its suitability for high-throughput screening applications. If this is not possible at the moment, including data on additional pharmacological compounds, e.g., modulators of voltage-gated Ca channels, which are key regulators of Ca signaling in beta cells of the pancreas would strengthen the study. (e.g., use voltage gated Ca channel blocker such as verapamil or nimodipine).

We thank the reviewer for this constructive suggestion. We would like to clarify that our pharmacological characterization was not limited to two compounds. In total, six compounds spanning two distinct ion channel targets were evaluated: the K-ATP channel modulators diazoxide, glimepiride, tolbutamide and nateglinide, and the TRPM3 modulators pregnenolone sulphate and isosakuranetin. This panel includes both agonists and antagonists across two mechanistically distinct targets, and we believe this is sufficient to demonstrate the platform's suitability for high-throughput compound screening in the context of a proof-of-concept study.

We nonetheless agree with the reviewer that extending the compound panel to include modulators of additional ion channel classes, such as voltage-gated Ca ^2+^ channel blockers like verapamil or nimodipine, would further demonstrate the versatility of the platform. We have added a statement to the Discussion explicitly identifying this as a valuable direction for future work.

(2) Testing another beta-cell line (e.g., INS-1 cells) would strengthen the manuscript.

We thank the reviewer for this suggestion. We agree that validating the platform using an additional β-cell line, such as INS-1 cells, would further broaden the applicability of the approach. We did indeed attempt experiments with INS-1 cells; however, the results were inconclusive due to cell quality issues at the time of testing, and we were unable to generate reliable data suitable for inclusion in the manuscript.

We would also like to emphasize that the primary aim of this study was to demonstrate the methodology of the high-throughput screening platform, rather than to provide a comprehensive cross-cell-line validation. In this context, the use of the well-established MIN6 β-cell line is sufficient to serve as a proof-of-principle demonstration of the platform's capabilities.

Nonetheless, we consider a systematic evaluation of INS-1 cells on this platform as an important and natural next step and have included this explicitly as a future perspective in the Discussion.

(3) I find the presentation of the results and analysis of Ca oscillation frequency and area under the curve excellent. Could you please provide more details on the analysis method used to quantify the frequency of glucose-induced Ca oscillation. If a custom script was used, sharing this information with the scientific community would be great.

We thank the reviewer for this positive feedback. We confirm that the Ca ^2+^ oscillation analysis was performed using a custom Python script (v3.10.11), the key steps of which are described in the Data Analysis section of the experimental procedures.

In line with our commitment to open and reproducible science, the script will be made publicly available upon acceptance of the manuscript, allowing the broader scientific community to apply, adapt, and build upon the analysis pipeline.

(4) Please move the Supplementary Figure to the main Figure 3. This will allow a direct comparison between Ca signals in spheroids and in single cell (2D cultures) under identical conditions.

We thank the reviewer for this suggestion. We have partially incorporated the supplementary figure into the main manuscript. The mean Ca ^2+^ response of 2D-cultured MIN6 cells at 20 mM glucose has been added as Figure 3D, enabling direct visual comparison with the spheroid data under identical stimulation conditions. The remaining panels of the supplementary figure — showing representative Ca ^2+^ traces across multiple glucose concentrations and the corresponding dose-response curves for peak frequency and area under the peaks in 2D monolayers — have been retained in the supplementary information, as their inclusion in the main figure would substantially increase its complexity. The figure legend has been updated accordingly.

(5) A direct comparison of insulin secretion between 3D cultured spheroids and 2D cultures should also be shown.

We thank the reviewer for this suggestion. We attempted to include a direct comparison of insulin secretion between 3D spheroids and 2D MIN6 monolayers; however, the 2D measurements proved unreliable for quantitative comparison. Insulin values in the 2D condition consistently exceeded the upper detection limit of the ELISA, and inter-well variability was too high to draw meaningful conclusions. We therefore chose not to include this comparison and instead present the Ca ^2+^ imaging data in Figure 3D as a functional readout enabling direct comparison between the two culture formats under identical stimulation conditions.

(6) The authors should further discuss the remaining effects of pregnenolone sulphate on insulin secretion.

We thank the reviewer for this comment. We would like to clarify that in our experimental setup, pregnenolone sulphate and isosakuranetin were applied simultaneously rather than sequentially. As a result, the incomplete inhibition of PS-induced Ca ^2+^ oscillations and insulin secretion observed in the presence of isosakuranetin may in part reflect a kinetic offset between the two compounds, whereby PS-induced TRPM3 activation and downstream signalling may have been initiated prior to the establishment of effective TRPM3 blockade by isosakuranetin. In addition, as noted in the Discussion, TRPM3 may not be the only molecular target of PS in these spheroids, and alternative signalling pathways may contribute to the residual insulin secretion observed in the presence of the antagonist. We have added a brief clarification to the Discussion to explicitly acknowledge the potential influence of this kinetic limitation on the interpretation of these results.

(7) Is it possible to collect 3D cultured spheroids after each experiment to measure for example intracellular insulin content or protein levels by Western blot.

Physical recovery of spheroids from the PEG hydrogel plates for downstream biochemical analysis, such as intracellular insulin content measurements or Western blot, would indeed be a meaningful addition to the platform's capabilities. We would like to note that the firm attachment of spheroids to the glass substrate, while essential for maintaining spheroid positioning during the extensive washing and liquid handling steps, does present a practical challenge for post-experimental recovery. Although we have successfully extracted spheroids of other cell types from comparable plate formats, reliable recovery of the MIN6 β-cell spheroids without compromising their structural integrity has not yet been achieved. We therefore identify the optimization of spheroid recovery as a valuable direction for future development of the platform.

(8) Ca signals in response to the application of glucose appears more robust in spheroids compared to single MIN6 cells. What are the possible mechanisms underlying this difference. Whole RNA-seq experiments would be one approach to identify differentially expressed genes in 2D versus 3D culture (this is maybe a whole project by itself). An alternative is to look by RT-qPCR analysis for key β-cell markers and genes encoding ion channel and ion channel subunits.

We thank the reviewer for this thoughtful comment. The more robust Ca ^2+^ signals observed in 3D spheroids compared to 2D monolayer cultures likely reflect several interconnected factors. First, and importantly, it should be noted that Ca ^2+^ measurements in 2D monolayer cultures typically represent an averaged signal across a large population of cells, which tends to obscure individual oscillatory events and reduce the apparent amplitude and regularity of Ca ^2+^ responses. In contrast, our 3D spheroid platform enables Ca ^2+^ measurements at the level of individual spheroids, allowing discrete oscillatory peaks to be resolved with much greater fidelity. Beyond this methodological distinction, the 3D architecture also promotes enhanced cell-to-cell communication, better recapitulation of in vivo β-cell coupling, and a more physiologically relevant microenvironment, all of which are likely to contribute to the improved oscillatory Ca ^2+^ dynamics observed.

We agree with the reviewer that elucidating the transcriptional underpinnings of these differences, through whole RNA-seq or targeted RT-qPCR analysis of key β-cell markers and genes encoding ion channel subunits, would be highly informative and represents an elegant approach to understanding the molecular basis of the observed functional improvements. As the reviewer rightly acknowledges, however, such experiments constitute a substantial research effort in their own right. We have added a statement to the Discussion identifying this as a valuable direction for future investigation, alongside the other platform development priorities already outlined.

(9) A more detailed discussion of the limitations of the platform and potential strategies to further improve this system would strengthen the manuscript.

We thank the reviewer for this constructive suggestion. In response, we have expanded the Discussion to provide a more comprehensive overview of the current limitations of the platform and the strategies we envision for future development. Specifically, the revised Discussion now addresses the following points:

First, the platform is currently optimized for the MIN6 insulinoma cell line, which differs from primary pancreatic beta cells in several important respects, including glucose sensitivity and secretory capacity. Initial attempts to adapt the protocol to hiPS-derived beta cells were unsuccessful, likely due to insufficient cellular viability following transfer to the micropatterned plate format. Optimizing the platform for use with primary beta cells or hiPS-derived beta cells is therefore identified as a priority for future development.

Second, while the current study demonstrates proof-of-concept pharmacological characterization using six compounds across two mechanistically distinct ion channel targets, extending the compound panel to include modulators of additional ion channel classes, such as voltage-gated Ca ^2+^ channel blockers, as well as validation using alternative insulinoma cell lines such as INS-1, would further demonstrate the versatility and generalizability of the platform.

Third, the introduction of perfusion-induced flow, which has been shown to improve viability, insulin secretory function, and morphological integrity of 3D beta-cell tissues under dynamic culture conditions, is identified as an additional avenue for optimization.

Fourth, while the current platform operates in a 96-well plate format, which already provides a substantial throughput of up to 1824 individual spheroid measurements per plate, adaptation to higher density plate formats such as 384-well or 1536-well plates would be a necessary step towards true high-throughput screening compatible with industrial drug discovery pipelines. Miniaturization of the hydrogel design and adaptation of the molding procedure to accommodate these formats therefore represents an important direction for future development.

Minor points:

(10) Please clarify the glucose concentration at which the MIN6 cells the cultured 3D beta cell spheroids were maintained overnight prior to the experiments.

Prior to the experiments, both 2D MIN6 cells and 3D MIN6 β-cell spheroids were maintained overnight in standard high-glucose DMEM (25 mM glucose), consistent with widely used MIN6 culture protocols. This information has been added to the Methods section.

(11) In Figure 3A, is the presented Ca trace derived from one single spheroid? Showing representative Ca traces (e.g., 5 i traces per condition) would show the reproducibility of the recordings.

We thank the reviewer for this suggestion. The trace shown in Figure 3A is indeed derived from a single representative spheroid. To address the concern regarding reproducibility, we refer the reviewer to the updated Figure 3C, which now displays the individual Ca ^2+^ response traces of all spheroids within a single well in response to 20 mM glucose stimulation. Grey lines represent individual spheroids, while the black line denotes the mean response. This panel illustrates not only the reproducibility of the oscillatory response across the imaged population but also highlights an important consequence of inter-spheroid variability: because individual spheroids oscillate asynchronously, their peaks cancel out when averaged, resulting in a mean trace that appears relatively flat and lacks the oscillatory features visible in individual recordings. We note that this same cancellation effect likely underlies the comparably flat mean response observed for 2D-cultured MIN6 cells in Figure 3D. Additionally, the difference in the initial response profile between 2D and 3D cultures may in part reflect the geometry of the hydrogel microenvironment. In 2D cultures, the glucose stimulus equilibrates rapidly and near-uniformly across the culture plane, potentially driving a more synchronized initial response and the early peak visible in Figure 3D. In contrast, the PEG hydrogel surrounding the 3D spheroids may act as a diffusion barrier, causing the stimulus to reach individual spheroids with variable delay and thereby further desynchronizing response onsets across the population. The figure legend has been updated accordingly.

(12) In Figure 3A, the potassium application bar looks a bit shifted. Please check and correct if necessary.

We thank the reviewer for carefully examining the figure. The apparent shift in the potassium application bar is not an error. In these experiments, glucose was added after an initial 10-minute baseline period, and the observed delay in the Ca ^2+^ response reflects two contributing factors. First, the glucose solution was pipetted at the top of the well, and diffusion to the level of the spheroids introduces a short lag before the stimulus reaches the cells. Second, the spheroid shown in Figure 3A was located at the outer edge of the well, where mixing is slower and the stimulus arrives with additional delay compared to centrally positioned spheroids. Together, these factors account for the offset between the start of the glucose application bar and the onset of the visible Ca ^2+^ response.

(13) Is the system also compatible with the ratiometric Ca imaging dye Fura-2?

The platform is indeed compatible with ratiometric Ca ^2+^ imaging using Fura-2. The glass-bottom plate format is a prerequisite for Fura-2 imaging due to the requirement for UV excitation at 340/380 nm, and the transparency of the PEG-based hydrogel ensures that the optical properties of the platform are fully compatible with this approach. Furthermore, the µCELL FDSS fluorescence plate imager used in this study supports dual-excitation ratiometric imaging, making it instrumentally compatible with Fura-2 without any additional hardware modifications.

(14) In Figure 4 legend: 100 µm diazoxide should be corrected to 100 µM diazoxide.

This has been addressed in the revised manuscript

(15) Please comment on the cost of spheroid generation compared with conventional 2D MIN6 cultures.

We thank the reviewer for this relevant question. The cost of spheroid generation using our platform is largely comparable to conventional 2D MIN6 cultures, with the primary additional expense being the specialized micropatterned PEG-based hydrogel plates. All other aspects of the workflow, including cell culture reagents, imaging consumables, and instrumentation, remain identical. It should be noted that providing a precise cost comparison is difficult, as the price of the hydrogel plates represents the dominant variable cost and is subject to change depending on production scale and supplier agreements. Nevertheless, we consider the platform to be cost-effective relative to alternative 3D culture systems, which often require more complex fabrication procedures or proprietary consumables.

**Reviewer #3 (Public review):**

Summary:

The primary objective of this study is to develop high-throughput screening assays utilizing homogeneous 3D cell cultures that more accurately replicate the intricate architecture and cellular communication found in tissues. The authors have chosen pancreatic islet β-cells as a model system to evaluate agents that modulate insulin release, which is particularly relevant given the increasing prevalence of diabetes mellitus-a significant global health concern. Moreover, the incorporation of human-based 3D spheroids, organoids, or organ-on-chip technologies into drug discovery protocols is essential for enhancing clinical translation, as candidate compounds identified using animal models have often demonstrated limited success in clinical settings.

Strengths:

This study was thoughtfully planned and skillfully carried out. The use of micropatterned hydrogels to observe 19 spheroids at once is an ingenious aspect, which has been effectively validated with Ca microfluorography. Overall, I found this investigation to be exceptionally well-executed and free from notable flaws, as the results clearly back up the conclusions. Additionally, the developed method achieved the proposed aims, providing a high-throughput format with 3D cultures. I believe this study deserves publication.

Weaknesses:

For an HTS assay, authors should incorporate the Z-factor.

We thank the reviewer for their valuable comment, which we have directly addressed in the revised version, as outlined below.

**Reviewer #3 (Recommendations for the authors):**

(1) The study is very well performed, but to support the claim of suitability for HTS, the Z-factor of the method should be reported.

We thank the reviewer for this helpful suggestion. In response, we have now included the Z′-factor in the manuscript to explicitly quantify assay performance and robustness. Specifically, the Z′-factor (0.6) has been added and discussed in the Results section, incorporated into the Discussion to contextualize assay suitability for high-throughput screening, and included in the Methods under “Statistical Analysis,” where its calculation is described.
